# Supplementary material for: A Global Screen for Assembly State Changes of the Mitotic Proteome by SEC-SWATH-MS
Source: Cell Syst. 2020 Feb 26;10(2):133–155.e6. doi: 10.1016/j.cels.2020.01.001 (PMC7042714; doi:10.1016/j.cels.2020.01.001)

Q9Y3E7 | CHMP3_HUMAN | CHMP3 CGI149 NEDF VPS24 CGI−149
Monomer MW [kDa]: 25.073 Monomer expected elution fraction: 52

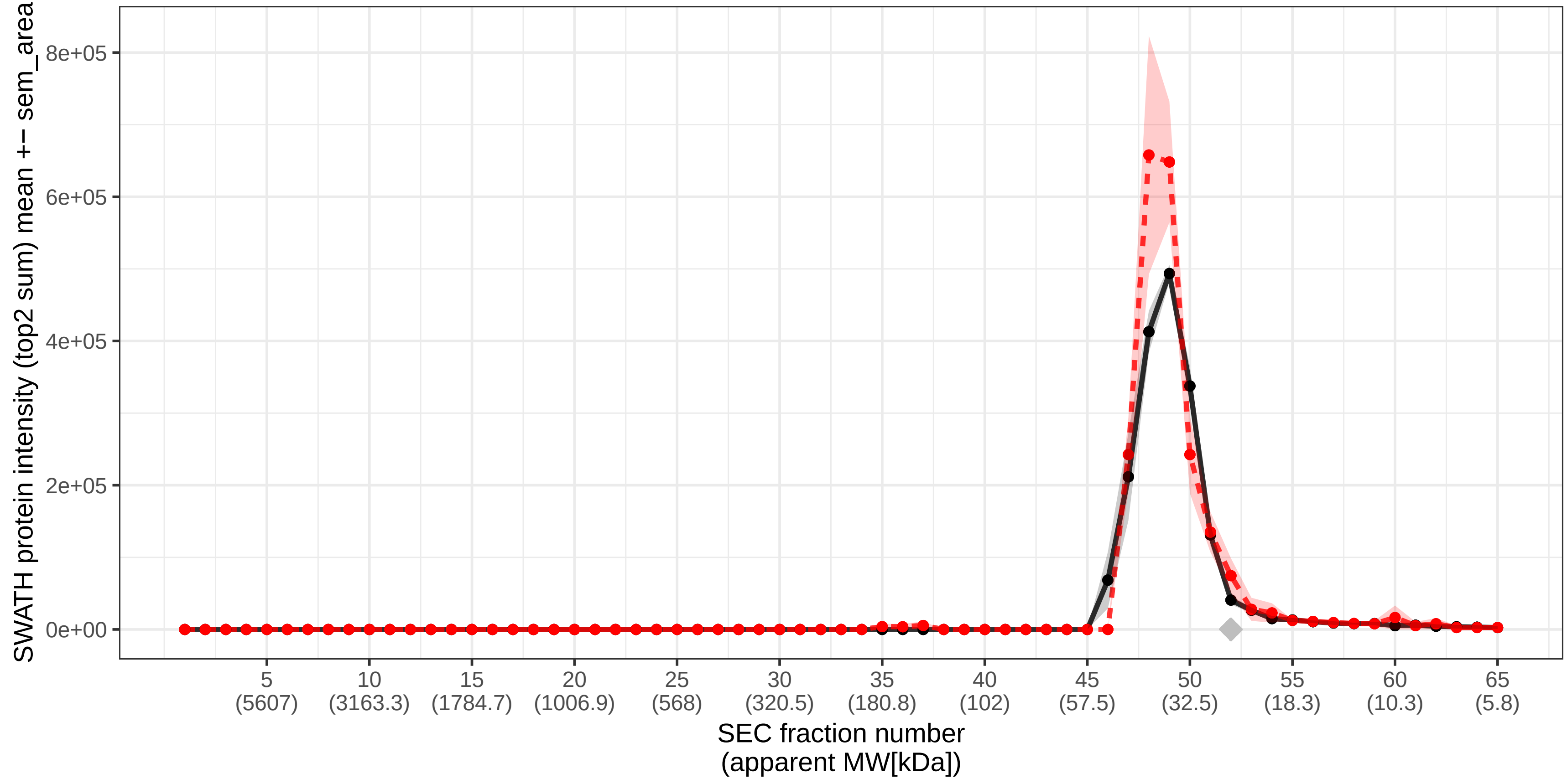

Supplement: Data S1. SEC-SWATH-MS Protein Chromatograms, Related to Figure 1 [file mmc6.zip › SECchrom_Q9Y3E7_CHMP3_HUMAN_CHMP3_CGI149_NEDF_VPS24_CGI-149.pdf]
